# Effects of phototherapy on muscle activity and pain in individuals with temporomandibular disorder: a study protocol for a randomized controlled trial

**DOI:** 10.1186/1745-6215-15-491

**Published:** 2014-12-16

**Authors:** Carolina Marciela Herpich, Ernesto Cesar Pinto Leal-Junior, Ana Paula Amaral, Juliana de Paiva Tosato, Igor Phillip dos Santos Glória, Marília Barbosa Santos Garcia, Bruno Roberto Borges Barbosa, Yasmin El Hage, Éric Edmur Camargo Arruda, Cid Ándre Fidelis de Paula Gomes, Monique Sampaio Rodrigues, Dowglas Fernando Magalhães de Sousa, Paulo de Tarso Camillo de Carvalho, Sandra Kalil Bussadori, Tabajara de Oliveira Gonzalez, Fabiano Politti, Daniela Aparecida Biasotto-Gonzalez

**Affiliations:** Student, Postgraduate Program in Rehabilitation Sciences, Center for Support to Research on Movement Analysis, University Nove de Julho (UNINOVE), Rua Profa Maria Jose Barone Fernandes, 300, São Paulo, SP 02117-020 Brazil; Teaching Staff Member , Postgraduate Program in Rehabilitation Sciences, Center for Support to Research on Movement Analysis, University Nove de Julho (UNINOVE), Rua Vergueiro, 235/249 - Liberdade, São Paulo, SP 01504-001 Brazil; Department of Physical Therapy, University Nove de Julho (UNINOVE), Av. Dr. Adolfo Pinto, 109. Água Branca, São Paulo, SP 05001-100 Brazil; Teaching Staff Member, Postgraduate Program in Rehabilitation Sciences, Center for Support to Research on Movement Analysis, University Nove de Julho (UNINOVE), Rua Profa Maria Jose Barone Fernandes, 300, São Paulo, SP 02117-020 Brazil

**Keywords:** phototherapy, temporomandibular joint disorder, physical therapy modalities

## Abstract

**Background:**

According to the International Association for the Study of Pain (IASP), the term temporomandibular disorder (TMD) regards a subgroup of orofacial pain, the symptoms of which include pain or discomfort in the temporomandibular joint, ears, masticatory muscles and neck on one or both sides, as well as joint sounds, limited mandibular movements or mandibular deviation and difficulties chewing. Phototherapy, such as low-level laser therapy (LLLT) and light-emitting diode (LED) therapy, is one of the resources used to treatment muscle pain. Thus, there is a need to investigate therapeutic resources that combine different wavelengths as well as different light sources (LLLT and LED) in the same apparatus.

The aim of the proposed study is to evaluate the effects of four different doses of phototherapy on pain, activity of the masticatory muscles (masseter and bilateral anterior temporal) and joint mobility in individuals with temporomandibular disorder. A further aim is to determine the cumulative effect 24 and 48 hours after a single session.

**Methods/Design:**

A placebo-controlled, double-blind, randomized, clinical trial will be carried out involving 72 women between 18 and 40 years of age with a diagnosis of myogenous TMD. The participants will then be randomly allocated to four groups totaling 18 individuals per group. Three groups will be submitted to a single session of phototherapy with different light sources, and one group will receive placebo therapy: Group A (2.62 Joules); Group B (5.24 Joules); Group C (7.86 Joules); and Group D (0 Joules). The following assessment tools will be administered on four separate occasions (baseline and immediately after, 24 h after and 48 h after phototherapy). Pain intensity will be assessed using the visual analog scale for pain, while pain thresholds will be determined using algometer, and electromyographic (EMG) analysis on the masseter and anterior temporal muscles.

**Discussion:**

The study will contribute to the practice of the evidence-based use of phototherapy in individuals with a myogenous TMD. Data will be published after the study is completed.

**Trial registration:**

This study is registered with the Brazilian Registry of Clinical Trials, NCT02018770, date of registration: 7 December 2013.

## Background

According to the International Association for the Study of Pain (IASP) [1], the term temporomandibular disorder (TMD) regards a subgroup of orofacial pain, the symptoms of which include pain or discomfort in the temporomandibular joint, ears, masticatory muscles and neck on one or both sides, as well as joint sounds, limited mandibular movements or mandibular deviation and difficulties chewing. Muscle pain is one of the most common and limiting clinical manifestations of this condition [2–5]. The main causes of TMD are trauma involving local tissues, chronic repetitive microtrauma, non-habitual use of the mandible and an increase in emotional stress [6]. With regard to prevalence, it is estimated that 20% of the population worldwide have TMD, but only 10 to 20% of affected individuals seek some form of treatment [7, 8]. Phototherapy, such as low-level laser therapy (LLLT) and light-emitting diode (LED) therapy, is one of the resources used by dentists and physiotherapists to treat TMD. Due to the low energy intensity and wavelengths capable of penetrating biological tissues, LLLT is believed to influence the synthesis, release and metabolism of numerous signaling substances involved in analgesia [9, 10]. This resource is reported to lead to an increase in the level of beta-endorphins, a reduction in bradykinin, a reduction in the release of histamine, an increase in lymphatic flow, reductions in swelling and pain-related substances, an increase in blood supply, a reduction in the duration of inflammation and the induction of muscle relaxation [11, 12]. LED therapy has been used for the same purposes and has demonstrated similar results, while offering the advantages of a lower cost and greater equipment durability [13, 14]. However, an exhaustive review of the literature has revealed no studies involving the use of phototherapy with different light sources (LLLT and LED) on the same device for application to the masseter and temporal muscles in individuals with myogenous TMD. Knowledge regarding the clinical effects of these forms of phototherapy can contribute to the indication of the best therapeutic resource for such patients. the use of therapeutic appliances that combine different sources of light may be advantageous, especially as devices with this feature began to be available in recent years commercially, making it necessary to research primarily on effects and the choice of best parameters, primarily analyzing immediate effect to later assess cumulative dose effects on the masticatory muscles in patients with TMD.

The aim of the proposed study is to evaluate the effects of four different doses of phototherapy (0, 2.62, 5.24 and 7.86 Joules) on pain, activity of the masticatory muscles (massester and bilateral anterior temporal) and joint mobility in individuals with temporomandibular disorder (TMD). A further aim is to determine the cumulative effect 24 and 48 hours after a single session. The hypothesis is that individuals with TMD submitted to phototherapy with different light sources (LLLT and LED) contained in a single device with a dose of 7.86 will experience a greater reduction in pain and change in electromyographic activity of the masticatory muscles in comparison to individuals submitted to lower doses and the effects of a single session of phototherapy will be maintained for 24 and 48 hours.

## Methods/Design

### Overview of research design

A double-blind, placebo-controlled, randomized, clinical trial is proposed to compare the effects of different doses of phototherapy in individuals with myogenous TMD. After screening, individuals with myogenous TMD (based on the Research Diagnostic Criteria for Temporomandibular Disorders (RDC/TMD) will be selected. The following evaluations and assessment tools will be administered: visual analog scale (VAS), algometry for pain, and electromyographic (EMG) analysis. Seventy-two individuals will be allocated to different groups (18 individuals per group) through a randomization process involving opaque envelopes containing cards stipulating one of the three following groups: A (2.62 Joules), B (5.24 Joules), C (7.86 Joules) and D (0 Joules). The study will be divided into three evaluation phases and a treatment phase:

#### Initial evaluation

The individuals who meet the eligibility criteria will undergo an initial evaluation in the following sequence: VAS, algometry for pain and EMG analysis on the masseter and anterior temporal muscles.

#### Treatment phase

Following randomization, the phototherapy will be carried out on the masseter and temporal muscles that are presenting pain.

#### Final evaluations

**Figure 1:**
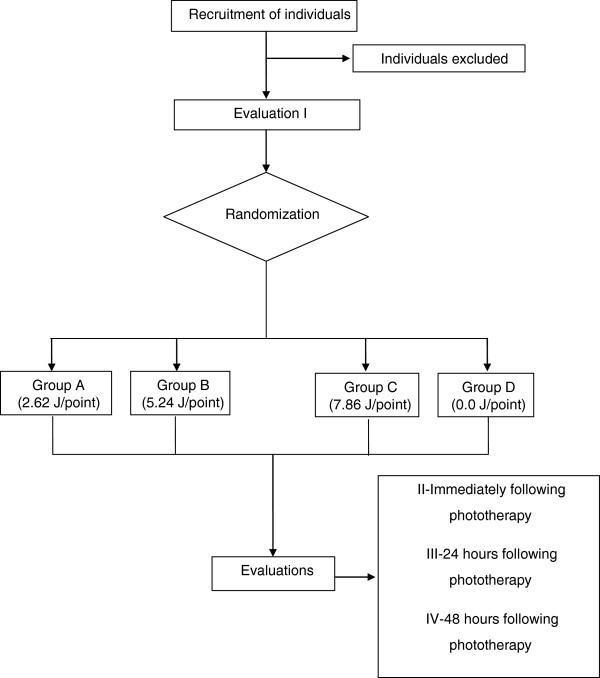
**Flow chart of experimental design.**

Immediately after, 24 hours after and 48 hours after the end of the treatment phase, the final evaluations will be performed, following the same sequence used in the initial evaluation (Figure 1).

### Blinding

The participants will be blinded to the allocation. A researcher will be responsible for the randomization process and programming of the phototherapy device based on the results of the randomization. A second researcher will perform the phototherapy and will be blinded to the doses and allocation of the participants. These researchers will not participate in the evaluations, which will be performed by a third researcher blinded to the allocation of the participants. The statistician will also be blinded to the allocation of the participants until the end of the statistical analyses.

### Inclusion criteria

All volunteers will have a diagnosis of myogenous TMD based on the RDC/TMD, with moderate to severe pain in the masseter and temporal muscles being rated by an expert dentist. To standardize the sample, the body mass index of all participants must be less than 25 kg/m2, as the amount of adipose tissue between the electrode and surface of the muscle can affect that reading of the electromyographic (EMG) signal [15]. All volunteers must also have an initial pain score greater than 3 points on the VAS [16].

### Exclusion criteria

The exclusion criteria will be missing teeth, the use of dentures, systemic or neuromuscular diseases, a history of trauma to the face or temporomandibular joint, history of temporomandibular joint luxation, currently undergoing orthodontic treatment, or currently undergoing medicinal treatment that can affect the musculoskeletal system (analgesic, anti-inflammatory agent or muscle relaxant).

### Procedures

The proposed study will follow the recommendations of the Consolidated Standards of Reporting Trials (CONSORT) statement to ensure greater transparency and quality of the findings. The participants will receive information on the objectives and the eligibility criteria of the study, and will subsequently provide informed consent. For eligible participants, the evaluator will collect the baseline data prior to randomization. This evaluator will be blinded to the allocation of the patients to the different groups. The following assessment tools will be applied: 1) RDC/TMD questionnaire for the diagnosis and inclusion of the participants; 2) EMG; 3) VAS for pain; and 4) an algometer. Detailed descriptions of each of these tools are offered below.

### Diagnosis of temporomandibular disorder

The RDC/TMD will be used for the diagnosis of TMD [17], which classifies individuals into three main groups: I) muscle disorder; II) disc displacement; and III) other joint conditions (Table 1). Each participant may be classified in one, two or three groups simultaneously. As pain will be the primary outcome of the study, all volunteers will need to have myofacial pain. Therefore, the RDC/TMD will be used to determine whether the volunteers meet this inclusion criterion. The RDC/TMD has two axes. Axis I consists of a clinical exam, which will be performed by a single examiner having previously undergone a training and calibration exercise in compliance with the specifications of the International RDC/TMD Consortium. This exam addresses the presence of spontaneous muscle and joint pain, maximum mouth opening, mandibular range of motion, joint sounds and pain sensitivity during mandibular movements or upon palpation of the muscles and/or joint. Average duration for the application of Axis I is 20 minutes. The volunteer will be seated in a chair with the trunk erect, back completely supported, feet on the floor and hands supported on the thighs, with the Frankfurt plane parallel to the ground. The examiner will be positioned in front of the volunteer. Axis II classifies individuals based on the degree of chronic pain, depression and nonspecific physical symptoms. This axis will be self-administered following the clinical exam in a well-lit, climate-controlled room with no time constraints.

**Table 1:** **Classification and diagnosis of temporomandibular disorder subgroups based on Research Diagnostic Criteria for Temporomandibular Disorders**

Group	Subgroup
I	A. Myofascial pain
	B. Myofascial pain with limited opening
	No group I diagnosis
II right	A. Disk displacement with reduction
	B. Disk displacement without reduction with limited opening
	C. Disk displacement without reduction without limited opening
	No group II diagnosis
II left	A. Disk displacement with reduction
	B. Disk displacement without reduction with limited opening
	C. Disk displacement without reduction without limited opening
	No group II diagnosis
II right	A. Arthralgia
	B. Osteoarthritis of temporomandibular joint syndrome (TMJ)
	C. Osteoarthritis of TMJ
	No group III diagnosis
II left	A. Arthralgia
	B. Osteoarthritis of TMJ
	C. Osteoarthritis of TMJ
	No group III diagnosis

### Electromyography

Electromyography (EMG) activity of the right and left masseter and anterior temporal muscles will be recorded using disposable, active surface electrodes (Ag/AgCl - Noraxon™- http://www.noraxon.com/*webcite* - 15770 N. Greenway-Hayden Loop, #100Scottsdale, AZ 85260. Phone: 480-443-3413) attached to the belly of the muscles in the region of greatest volume determined during moderate jaw clenching. The skin surface will be cleaned with cotton soaked in 70% alcohol to diminish impedance [15]. A metallic rectangular reference electrode measuring 3 × 2 cm coated with Lectron II conductive gel (Pharmaceutical Innovations™ http://www.pharminnovations.com*webcite* - 897 Frelinghuysen Ave Newark, NJ 07114 (973) 242–2903) to enhance the conduction capacity and impede interference from external noise will be attached to the left wrist of each participant.

The EMG signals will be collected with the aid of an eight-channel electromyograph (830 C module, EMG System do Brazil Ltda™)- http://www.emgsystem.com.br*webcite* - Rua Porto Principe, 50 - Vila RubiCEP12245-572-São José dos Campos/SP. Phone: 55 12 3922-4069/55 12 3942–4736)) using a band-pass filter with a cutoff frequency of 20 to 1000 Hz and common-mode rejection ratio >120. All data will be collected and processed using 16-bit analog-digital converter (EMG System do Brazil Ltda™ - http://www.emgsystem.com.br*webcite* - Rua Porto Principe, 50 - Vila RubiCEP12245-572-São José dos Campos/SP. Phone: 55 12 3922-4069/55 12 3942–4736))) with a sampling frequency of 2 kHz. The signal capturing module will be connected to a standard laptop computer with a compatible data acquisition program.

The volunteer will be seated in a chair, with the back completely supported, eyes open, feet parallel on the a rubber mat and arms supported on the thighs. The exam will be carried out under three conditions: 1) in the normal postural position with the mandible at rest; 2) during maximum voluntary contraction (MVC) with Parafilm M™ placed between the teeth; and 3) during maximum habitual contraction (MHC) with no material placed between the teeth. In the normal postural position, the volunteer will remain with the mandible relaxed, teeth apart and lips in contact for 15 s. For the MVC, the volunteer will be instructed to position Parafilm M™ between the upper and lower premolars, first molars and second molars bilaterally and perform isometric contraction for 5 s following a verbal command. For the MHC, the same procedure will be performed without the use of M™ for 5 s. Under each condition, three readings will be made with a 2-minute rest interval between readings to avoid fatigue.

### Electromyography signal processing

The root mean square (RMS), median frequency, asymmetry index and co-contraction index will be used as the parameters for the interpretation of the signal processing. The signals will be processed using specific routines developed with the aid of the Matlab program, version 7.1 (The MathWorks Inc., Massachusetts, USA). The RMS of the signal (expressed in μV) obtained at rest and during MHC will be normalized by the largest RMS obtained during three readings of MVC (μmV/μV × 100: %MVC). With the mandible at rest, the RMS will be calculated for the entire 15 s of the signal collected. Data on MHC will be analyzed based on Ferrario *et al*. [18], with the first and last second of the raw EMG signal being discarded and the RMS being calculated using a 25-ms moving window without overlap for the 3 s of signal selected.

The overlap percentage coefficient (OPC, unit: %) described by Ferrario *et al*. [18] will be used to evaluate the distribution of muscle activity in time and the amplitude determined by the occlusion. Symmetric distribution of muscle activity determined by the OPC will be analyzed considering variability between 0% (absence of symmetry) and 100% (perfect symmetry). Lateral excursion (potential given by imbalanced contractile activities of the contralateral masseter and temporal muscles) will be evaluated using the torque coefficient (TC, unit: %) considering variability between 0% (absence of lateral excursion force) and 100% (maximum lateral excursion force).

The EMG signal of the paired muscles under each situation recorded during MHC will be compared by calculating a percentage of OPC and TC. Mean normalized EMG activity (%MVC) of the right and left masseter and temporal muscles will be calculated and used for comparisons among evaluation times (pre-treatment, immediate post-treatment and late post-treatment).

### Visual analog scale

A visual analog scale (VAS) allows the quantification of pain intensity. This scale consists of a straight line measuring 10 cm in length, with ‘absence of pain’ written at one end and ‘worst pain ever felt’ written at the other end. The volunteer will be instructed to make a perpendicular line between the two extremes that represents the pain level he/she is feeling at the time [19].

### Algometry

A digital algometer (DD-200, Instrutherm™) will be used to evaluate pain through the application of pressure. For such, the volunteer will be seated in a chair, trunk erect, back completely supported, feet on floor and hand on thighs, respecting the Frankfurt plane. The researcher will position the algometer and apply gradual pressure at three points over the masseter muscle (upper, middle and lower), three points over the temporal muscle (posterior, middle and anterior) and over the lateral pole (bilaterally) based on the findings of the clinical exam (palpation) of the RDC/TMD. Pressure will be applied to all points until the volunteer reports feeling pain. The value registered on the display of the algometer will then be recorded. If the volunteer does not feel pain, maximum pressure will not exceed 4 Kgf [20]. Pressure will be applied using the rubber tip measuring 1 cm^2^ in direct contact with the skin at the ‘fast’ velocity of the ‘peak hold’ function (specification of the DD-200 digital algometer, Instrutherm™). Prior to the readings, the algometer will be applied to the volunteer’s arm to familiarize him/her with the procedure. The volunteer will be instructed to raise his/her hand to indicate the exact instant of pain, at which point the researcher will cease to apply the pressure. The algometer will be applied only once to each of the aforementioned points and a 30-s rest interval will be given between readings.

### Phototherapy

The portable PainAway™ nine-diode cluster (Multi Radiance Medical®, Solon, OH, USA) will be employed. This system has one 905 nm laser diode, four 875 nm LED diodes and four 670 nm LED diodes. The aperture size of device is 4 cm^2^ (Table 2).

**Table 2:** **Phototherapy parameters**

Number of super-pulsed lasers	1 Super-pulsed laser
Wavelength (nm)	905
Frequency (Hz)	1000
Average optical output (mW)	0.9
Peak power (W)	8.5
Dose (J) total per group (20s, 40s, 60s)	0.018; 0.036; 0.054
Spot size (cm2)	0.4
Number of red LEDs	4 Red
Wavelength (nm)	640(±10 nm)
Frequency (Hz)	2
Average optical output (mW)-each	15
Dose (J) each emitter per group (20s, 40s, 60s)	0.3; 0.6; 0.9
Dose (J) total per group (20s, 40s, 60s)	1.2; 2.4; 3.6
Spot size (cm2)-each	0.9
Number of infrared LEDs	4 infrared
Wavelength (nm)	875(±10 nm)
Frequency (Hz)	16
Average optical output (mW)-each	17.5
Dose (J) each emitter per group (20s, 40s, 60s)	0.35; 0.70; 1.05
Dose (J) total per group (20s, 40s, 60s)	1.4; 2.8; 4.2
Spot size (cm2)-each	
Magnetic field (mT)	35
Treatment time (s)	20; 40 or 60
Aperture of device (cm^2^)	4
Total delivered energy (J) per point	2.62; 5.24 or 7.86
Total delivered energy (J) per individual	26.20; 52.40 or 78.60

For the blinding of the participants, the phototherapy equipment with the different light sources has two identical application hand pieces provided by the manufacturer - one with an active tip and the other for placebo applications (does not emit energy), but both with the same sound device. The tips will be denominated X and Y by a researcher who will not participate in the application or evaluations. The researchers who will administer the phototherapy procedures and those in charge of the evaluations will be blinded to which hand piece has the active effect and which is for the placebo application. The hand pieces will only be identified at the end of the data collection process. Table 2 displays the doses and application times for each group.

Phototherapy with the different light sources will be applied to two points of the masseter muscle bilaterally and three points on the temporal muscle bilaterally (totaling 10 application points), based on the results of the clinical exam performed using the RDC/TMD. The same points will be evaluated using an algometer.

Phototherapy will be applied by one of the researchers in an isolated room with only the researcher and the volunteer present. The researcher will receive the pre-programmed hand piece. Both the researcher and volunteer will be wearing eye protection equipment. The volunteer will be in the supine position on a cot with lower limbs supported on a roll of foam rubber measuring 20 cm in diameter and head supported on a pillow to provide a comfortable resting posture. Phototherapy will be applied using the point technique in contact with the skin with a beam area of 4 cm^2^ (device aperture). The placebo group will be submitted to the same procedures as the groups submitted to phototherapy. Only the coordinator will have knowledge regarding the different phototherapy and placebo applications. At the end of the study, the individuals in the placebo group will be submitted to treatment with active phototherapy.

### Sample size

The sample size was calculated considering α = 0.05 (5% chance of a type I error), 1-β = 0.95 (% of sample power) and data from a visual analog scale (VAS) reported in the study by Pereira *et al*. [21]. The minimum number for each group was determined to be 15 individuals, to which 20% was added to compensate for possible dropouts during the study (total: 18 individuals per group). This calculation was performed using the G*Power software program [22]. Seventy-two individuals will be allocated to different groups (18 individuals per group) through a randomization process involving opaque envelopes containing cards stipulating one of the three following groups: A (2.62 Joules), B (5.24 Joules), C (7.86 Joules) and D (0 Joules).

### Data analysis

Pain will be the primary outcome and will be determined using a VAS and algometer. EMG will be the secondary outcome. The independent variables will be the different light sources used for phototherapy. The dependent variables will be derived from the pre-treatment and post-treatment evaluations. Intra-group comparisons will be performed using repeated-measures analysis of variance (ANOVA or Friedman’s test), followed by either the Student’s t-test or Wilcoxon’s test. Inter-group comparisons will be performed using either one-way ANOVA or the Kruskal-Wallis test. The choice of tests will be determined by the distribution (normal or non-normal) of the data. The level of significance will be set to 5% (*P* <0.05). All comparisons and statistical analyses will be performed using the SPSS program, version 13.0 (Chicago, IL, USA). Cohen’s d will be employed to determine the clinical effect size of the proposed therapies, with the interpretation of the coefficients based on the classification established by Cohen [23]: 0.2 = small effect; ≥0.5 = moderate effect; ≥0.8 = large effect.

### Ethics and data security

The proposed randomized, placebo-controlled, double-blind trial received approval from the Human Research Ethics Committee of the University Nove de Julho (Sao Paulo, Brazil) under process number 18032013.4.0000.5511 dated 6 June 2013. All individuals will be asked to provide written, informed consent prior to randomization, using standard forms. The trial is registered with the World Health Organization under Universal Trial in ClinicalTrials.gov under the number NCT02018770.

## Discussion

The findings are expected offer scientific evidence regarding the importance of an effective conservative treatment option for patients with TMD and assist physiotherapists in the clinical decision-making process when working with such patients. Although a number of studies in the literature have addressed different forms of treatment for TMD [24–27] including phototherapy [28, 29], gaps in knowledge remain in clinical practice regarding the use of different light sources (LLLT and LED), as well as the most effective, evidenced-based doses.

In a systematic review, Melis *et al*. [30] found considerable methodological differences among studies, especially with regard to the number of applications, duration and characteristics (wavelength and frequency) of LLLT, demonstrating the lack of standardized guidelines for effective treatment. Thus, there is a need for further in-depth investigations regarding the use of phototherapy for the treatment of myogenous TMD [24–27]. Pain is the main symptom of this condition and muscle and joint involvement are the most prevalent in the population [31], leading to limitations with regard to activities of daily living, such as eating, speaking, yawning and smiling, as well as a reduction in quality of life [32].

The study will contribute to the practice of evidence-based use of phototherapy in individuals with a myogenous TMD, and the effects of specific phototherapy parameters can help optimize treatment strategies for reducing and even eliminating the signs and symptoms of myogenous TMD. Data will be published after the study is completed.

## Trial status

The proposed study is currently in the recruitment phase, and individuals are being diagnosed with TMD using the RDC-TMD questionnaire.

## Abbreviations

EMG: electromyography
LED: light emitting diode
LLLT: low-level laser therapy
MHC: maximum habitual contraction
MVC: maximum voluntary contraction
OPC: overlap percentage coefficient
RDC/TMD: Research Diagnostic Criteria for Temporomandibular Disorders
RMS: root mean square
ROM: range of motion
TC: torque coefficient
TMD: temporomandibular disorder
VAS: visual analog scale.

## References

[1] IASP (1994). Classification of Chronic Pain: Descriptors Of Chronic Pain Syndromes And Definitions Of Pain Terms.

[2] Truelove EL, Sommers EE, LeResche L, Dworkin SF, Von KM (1992). Clinical diagnostic criteria for TMD. New classification permits multiple diagnoses. J Am Dent Assoc.

[3] Plesh O, Sinisi SE, Crawford PB, Gansky SA (2005). Diagnoses based on the research diagnostic criteria for temporomandibular disorders in a biracial population of young women. J Orofac Pain.

[4] Orlando B, Manfredini D, Bosco M (2006). Efficacy of physical therapy in the treatment of masticatory myofascial pain: a literature review. Minerva Stomatol.

[5] Gonçalves DA, Dal Fabbro AL, Campos JA, Bigal ME, Speciali JG (2010). Symptoms of temporomandibular disorders in the population: an epidemiological study. J Orofac Pain.

[6] Sarlani E (2003). Diagnosis and treatment of orofacial pain. Braz J Oral Sci.

[7] Pedroni CR, Oliveira AS, Guaratini MI (2003). Prevalence study of signs and symptoms of temporomandibular disorders in university students. J Oral Rehabil.

[8] Nassif NJ, Al-Salleeh F, Al-Admawi M (2003). The prevalence and treatment needs of symptoms and signs of temporomandibular disorders among young adult males. J Oral Rehabil.

[9] Marini I, Gatto MR, Bonetti GA (2010). Effects of superpulsed low-level laser therapy on temporomandibular joint pain. Clin J Pain.

[10] Andrade TNC, Frade JC (2008). Estudo comparativo entre os efeitos de técnicas de terapia manuais isolados e associada à laserterapia de baixa potência sobre a dor em pacientes com disfunção temporomandibular. Rev Gauch Odontol.

[11] Da Cunha LA, Firoozmand LM, Da Silva AP, Camargo SE, Oliveira W (2008). Efficacy of low-level laser therapy in the treatment of temporomandibular disorder. Int Dent J.

[12] Emshoff R, Bösch R, Pümpel E, Schöning H, Strobl H (2008). Low-level laser therapy for treatment of temporomandibular joint pain: a double-blind and placebo-controlled trial. Oral Surg Oral Med Oral Pathol Oral Radiol Endod.

[13] Bastos JLN, Lizarelli RFZ, Parizotto NA (2009). Comparative study of laser and LED systems of low intensity applied to tendon healing. Laser Phys.

[14] Yeh NG, Wu CH, Cheng TA (2010). Light-emitting diodes-their potential in biomedical applications. Ren Sust Energ Rev.

[15] De Luca CJ (1997). The use of surface electromyography in biomechanics. J Appl Biomech.

[16] La Touche R, Fernández-De-Las-Peñas C, Fernández-Carnero J, Escalante K, Angulo-Díaz-Parreño S, Paris-Alemany A, Cleland JA (2009). The effects of manual therapy and exercises directed at the cervical spine on pain sensitivity in patients with myofascial temporomandibular disorders. J Oral Rehabil.

[17] Dworkin SF, Leresche L (1992). Research diagnostic criteria for temporomandibular disorders: review, criteria, examinations and specifications, critique. J Craniomandib Disord.

[18] Ferrario VF, Sforza C, Colombo A, Ciusa V (2000). A electromiographic investigation of masticatory muscles symmetry in normo-occlusion subjects. J Oral Rehabil Oxford.

[19] Ferreira-Valente MA, Pais-Ribeiro JL, Jensen MP (2011). Validity of four pain intensity-rating scales. Pain.

[20] Ylinen J, Nykanen M, Kautiainen H, Hãkkinem A (2009). Evaluation of repeatability of pressure algometry on the neck muscles for clinical use. Man Ther.

[21] Pereira TS, Flecha OD, Guimarães RC, De Oliveira D, Botelho AM, Ramos Glória JC, Aguiar Tavano KT (2014). Efficacy of red and infrared lasers in treatment of temporomandibular disorders–a double blind, randomized, parallel clinical trial. Cranio.

[22] Faul F, Erdfelder E, Lang AG, Buchner A (2007). G*power 3: a flexible statistical Power analysis program for the social, behavioral, and biomedical sciences. Behav Res Methods.

[23] Cohen J (1988). Statistical Power Analysis For The Behavioral Sciences.

[24] Rodrigues-bigaton D, Almeida AFN, Berni KCS, Pedroni CR, Gonçalves RN, Bérzin F (2008). Use of different electrical stimulations for treating pain in women with temporomandibular disorders. Rev Bras Fisioter.

[25] Gomes NCMC, Berni-schwarzenbeck KCS, Packer AC, Rdrigues-Bigaton D (2012). Effect of cathodal high-voltage electrical stimulation on pain in women with TMD. Rev Bras Fisioter.

[26] Arruda EEC, Amaral AP, Politti F, Hage YE, Gomes CAFP, Cesar GM, Gonzalez TO, Biasotto-Gonzalez DA (2012). Immediate effects of mandibular mobilization on static balance in individuals with temporomandibular disorder pilot study. Clin Exp Med Lett.

[27] Amaral AP, Politti F, Hage YE, Arruda EEC, Amorin CF, Biasotto-Gonzalez DA (2013). Immediate effect of nonspecific mandibular mobilization on postural control in subjects with temporomandibular disorder: a single-blind, randomized, controlled clinical trial. Braz J Phys Ther.

[28] Bjordal JM, Couppé C, Chow RT, Tunér J, Ljunggren EA (2003). A systematic review of low-level laser therapy with location-specific doses for pain from chronic joint disorders. Aust J Physiother.

[29] Fikácková H, Dostálová T, Navrátil L, Klaschka J (2007). Effectiveness of low-level laser therapy in temporomandibular joint disorders: a placebo-controlled study. Photomed Laser Surg.

[30] Melis M, Di Giosia M, Zawawi KH (2012). Low level laser therapy for the treatment of temporomandibular disorders: a systematic review of the literature. J Craniomand Pract.

[31] Manfredini D, Winocur E, Ahlberg J, Guarda-Nardini L, Lobbezoo F (2010). Psychosocial impairment in temporomandibular disorders patients. RDC/TMD axis II findings from a multicentre study. J Dent.

[32] Turp JC, Motschall E, Schindler HJ, Heydecke G (2007). In patients with temporomandibular disorders, do particular interventions influence oral health-related quality of life? A qualitative systematic review of the literature. Clin Oral Implants Res.

